# Nanopore metagenomic sequencing of influenza virus directly from respiratory samples: diagnosis, drug resistance and nosocomial transmission, United Kingdom, 2018/19 influenza season

**DOI:** 10.2807/1560-7917.ES.2021.26.27.2000004

**Published:** 2021-07-08

**Authors:** Yifei Xu, Kuiama Lewandowski, Louise O Downs, James Kavanagh, Thomas Hender, Sheila Lumley, Katie Jeffery, Dona Foster, Nicholas D Sanderson, Ali Vaughan, Marcus Morgan, Richard Vipond, Miles Carroll, Timothy Peto, Derrick Crook, A Sarah Walker, Philippa C Matthews, Steven T Pullan

**Affiliations:** 1 Nuffield Department of Medicine, University of Oxford, Oxford, United Kingdom; 2 NIHR Oxford Biomedical Research Centre, University of Oxford, Oxford, United Kingdom; 3 Public Health England, National Infection Service, Porton Down, Salisbury, United Kingdom; 4 Department of Infectious Diseases and Microbiology, Oxford University Hospitals NHS Foundation Trust, John Radcliffe Hospital, Oxford, United Kingdom; 5 Nuffield Department of Medicine, Peter Medawar Building for Pathogen Research, University of Oxford, Oxford, United Kingdom

**Keywords:** influenza, Nanopore, metagenomics, diagnosis, antiviral drug resistance, genetic diversity, nosocomial transmission, respiratory viruses

## Abstract

**Background:**

Influenza virus presents a considerable challenge to public health by causing seasonal epidemics and occasional pandemics. Nanopore metagenomic sequencing has the potential to be deployed for near-patient testing, providing rapid infection diagnosis, rationalising antimicrobial therapy, and supporting infection-control interventions.

**Aim:**

To evaluate the applicability of this sequencing approach as a routine laboratory test for influenza in clinical settings.

**Methods:**

We conducted Oxford Nanopore Technologies (Oxford, United Kingdom (UK)) metagenomic sequencing for 180 respiratory samples from a UK hospital during the 2018/19 influenza season, and compared results to routine molecular diagnostic standards (Xpert Xpress Flu/RSV assay; BioFire FilmArray Respiratory Panel 2 assay). We investigated drug resistance, genetic diversity, and nosocomial transmission using influenza sequence data.

**Results:**

Compared to standard testing, Nanopore metagenomic sequencing was 83% (75/90) sensitive and 93% (84/90) specific for detecting influenza A viruses. Of 59 samples with haemagglutinin subtype determined, 40 were H1 and 19 H3. We identified an influenza A(H3N2) genome encoding the oseltamivir resistance S331R mutation in neuraminidase, potentially associated with an emerging distinct intra-subtype reassortant. Whole genome phylogeny refuted suspicions of a transmission cluster in a ward, but identified two other clusters that likely reflected nosocomial transmission, associated with a predominant community-circulating strain. We also detected other potentially pathogenic viruses and bacteria from the metagenome.

**Conclusion:**

Nanopore metagenomic sequencing can detect the emergence of novel variants and drug resistance, providing timely insights into antimicrobial stewardship and vaccine design. Full genome generation can help investigate and manage nosocomial outbreaks.

## Introduction

Influenza A viruses (IAV) are enveloped viruses of the *Orthomyxoviridae* family, with a segmented, ca 13 kb RNA genome [1,2]. IAV can cause both seasonal epidemics and occasional pandemics, presenting a considerable challenge to public health [3]. Seasonal epidemics are estimated to cause half a million deaths globally each year, primarily among young children and the elderly [4]. Estimates suggest a future pandemic could infect 20% to 40% of the world population and cause over 30 million deaths within 6 months [5,6]. Tracking and characterisation of circulating influenza viruses, in both human and animal populations, is critical to provide early warning of the emergence of novel variants with high virulence and to inform vaccine design.

Direct-from-sample metagenomic sequencing can potentially identify all viral and bacterial pathogens within an individual clinical sample. The genomic information generated can comprehensively characterise the pathogens and enable investigation of epidemiology and transmission. Oxford Nanopore Technologies (ONT; Oxford, United Kingdom (UK)) is a third generation sequencing technology that can generate long-read data in real-time, which has been successfully applied in the real-time surveillance of Ebola, Zika, and Lassa fever outbreaks [7-9]. Nanopore metagenomic sequencing has the potential to be deployed for near-patient testing, providing rapid and accurate diagnosis of infection [10], informing antimicrobial therapy [11], and supporting interventions for infection prevention and control [12]. We have recently demonstrated proof-of-principle for a direct-from-sample Nanopore metagenomic sequencing protocol for influenza viruses with 83% sensitivity and 100% specificity compared with routine clinical diagnostic testing [13].

Here we describe Nanopore metagenomic sequencing directly from clinical respiratory samples at a UK hospital during the 2018/19 influenza season, evaluating the applicability of this approach in a routine laboratory as a test for influenza, and investigating where further optimisation is still required before the assay can be deployed in clinical practice. We assessed the performance of this experimental protocol in comparison with routine clinical laboratory tests, and used the influenza sequence data to investigate drug resistance, genetic diversity, and nosocomial transmission events, demonstrating the diverse benefits that can be gained from a metagenomic approach to diagnostics.

## Methods

### Sample collection from clinical diagnostic laboratory

Residual material was collected from anonymised throat swabs, nasal swabs, and nasopharyngeal aspirates that had been submitted to the clinical diagnostic laboratory at the Oxford University Hospitals National Health Service (NHS) Foundation Trust during the 2018/19 influenza season.

Prior to metagenomic sequencing, samples had been tested in the diagnostic laboratory based on a standard operating protocol using either Xpert Xpress Flu/RSV assay (Cepheid, Sunnyvale, California, United States (US)), that detects influenza A/B and respiratory syncytial virus (RSV), or BioFire FilmArray Respiratory Panel (RP) 2 assay (BioFire Diagnostics, Salt Lake City, UT, US) that detects a panel of viral and bacterial respiratory pathogens). Xpert reports a quantitative diagnostic result (quantification cycle (Cq) value) for the detected pathogen, while BioFire RP2 reports a binary result (pathogen detected or not detected). The diagnostic laboratory routinely applies the BioFire RP2 assay to samples from a defined subgroup of patients most at risk of severe, complicated, or atypical disease (those who are immunocompromised, under the care of infection and respiratory teams, or admitted to critical care units).

### Sample selection for Nanopore metagenomic sequencing

The first laboratory diagnosis of influenza in our hospital laboratory in the 2018/19 season was made on 30 October 2018, and our sample collection ran until 5 February 2019. During this period, 1,789 respiratory samples were submitted to the diagnostic laboratory and tested by Xpress Flu/RSV assay, of which 213 were positive for IAV (11.9%); 752 samples were tested by BioFire FilmArray RP2 assay, of which 27 were positive for IAV (3.6%).

Samples positive for influenza (n = 90; based on results from Xpress Flu/RSV) and samples negative for influenza (n = 90; based on results from BioFire RP2 assay) were selected for Nanopore metagenomic sequencing as described below (Supplemental Figure S1 and Table S1A).

#### Selection of influenza-positive samples

**Figure 1:**
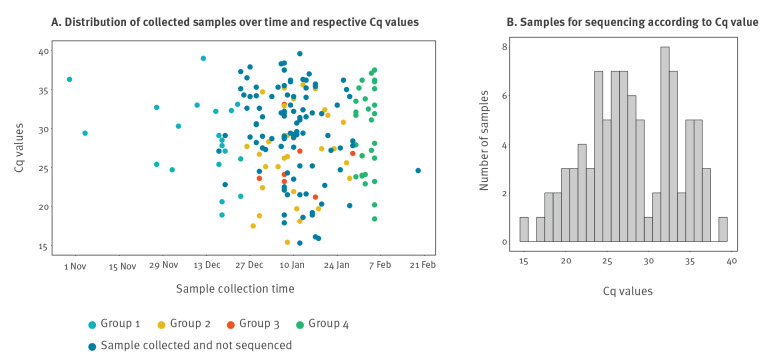
Nanopore metagenomic sequencing of respiratory samples submitted to the clinical diagnostic laboratory at the Oxford University Hospitals NHS Foundation Trust, United Kingdom, 2018/19 influenza season (n = 90 influenza-positive samples)

The 90 influenza-positive samples belonged to four groups (Figure 1A and Supplemental Figure S1). Group 1 included the first 20 positive samples of the influenza season, from 30 October to 24 December 2018. Group 2 (n = 33) consisted of randomly selected samples from the intervening period between Group 1 and 4. Group 3 (n = 8) was made up of samples from a putative transmission cluster on the infectious diseases ward between 29 December 2018 and 29 January 2019. Group 4 (n = 29) was composed of all influenza positive samples from the week beginning 30 January 2019 immediately before the onset of sequencing in this study, to represent consecutive samples from a single week at the peak of the influenza season.

Cq: quantitative cycle; NHS: National Health Service.

A. Distribution of 90 influenza-positive samples selected for Nanopore metagenomics sequencing (Oxford Nanopore Technologies, Oxford, United Kingdom) among total influenza-positive samples collected. Group 1 (n = 20): the first 20 positive samples of the influenza season. Group 2 (n = 33): randomly selected samples from the intervening period between Group 1 and 4. Group 3 (n = 8): samples from a putative transmission cluster on the infectious diseases ward. Group 4 (n = 29): all influenza positive samples from the week beginning 30 January 2019 immediately before the onset of sequencing in this study.

B. Histogram of Cq values of 90 influenza-positive samples selected for sequencing (Cq-value range: 15−39; mean: 28). Cq values were derived from Xpert Xpress Flu/RSV assay (Cepheid, Sunnyvale, California, United States) in the clinical diagnostic laboratory.

#### Selection of influenza-negative samples

Two groups with a combined total of 90 influenza-negative samples were considered. The first group comprised 55 samples positive for one of the following viruses: seasonal coronavirus (n = 10), rhino/enterovirus (n = 20), human metapneumovirus (HMPV; n = 5), parainfluenza (PIV; n = 10), and RSV (n = 10). Among them, one was positive for both RSV and HMPV, another was positive for PIV, HMPV, and adenovirus. The second group consisted of 35 samples negative for all pathogens tested by the BioFire RP2.

### Sample processing for sequencing

The 180 samples selected for sequencing were processed as described in detail previously [13]. Briefly, viral transport media from samples, spiked with 10^4^ genome copies per mL of Hazara virus as a positive internal control, was centrifuged to remove bacteria and cellular debris. Total nucleic acid was extracted, DNA was removed enzymatically and the remaining RNA was randomly reverse transcribed and amplified using sequence independent single primer amplification (SISPA). The resulting cDNA was prepared for Nanopore sequencing.

### Nanopore library preparation and sequencing

Multiplex sequencing libraries were prepared using 200 fmol of cDNA from every six samples as input to the SQK-LSK109 Kit, barcoded using the EXP-NBD104 Native barcodes (ONT). One Library was sequenced on FLO-MIN106 flow cells on a GridION device (ONT), with sequencing proceeding for 48 h. Samples for which sequencing did not produce reads of internal control Hazara genome were associated with low quantities of total cDNA and so were repeated with the addition of 5 μg linear polyacrylamide carrier (Thermo Fisher, Waltham MA, United States) to the AVL lysis buffer (Qiagen, Hilden, Germany).

The sample processing and library preparation time was 8 h, the sequencing time was 48 h, and thus total turnaround time for each sample was < 72 h. With a team of three members, we prepared the sequencing libraries for 180 samples within 9 days and completed the sequencing for the 90 influenza-positive samples within 12 days of commencing sequencing (Supplemental Figure S1). The turnaround time is dependent on the capacity of the laboratory, the hardware being used (e.g. MinION or GridION) and the time over which data are collected from the flow cells.

### Genomic analysis

Nanopore reads were base-called using Guppy v3 (ONT). Human reads were removed using CRuMPIT workflow [14]. In order to minimise the number of misclassified reads and to allow accurate identification of viral species, stringent demultiplexing was performed, which required the same barcode to be present at both ends of each read, using Porechop (v0.2.2, https://github.com/rrwick/Porechop). Reads were taxonomically classified against the RefSeq database using Centrifuge v1.0.3 [15]. Reads were then mapped using Minimap2 [16] to a reference genome for each viral species identified by Centrifuge. In order to optimise the selection of reference sequence, a draft consensus sequence for each viral species was generated using a simple majority voting method through selecting the most abundant base at each position. This resulting draft consensus sequence was investigated by Basic Local Alignment Search Tool (BLAST) against a custom viral database containing genomes of influenza, coronavirus, HMPV, RSV, PIV, and enterovirus, to identify the closest reference genomes. Reads were then mapped against the identified reference genome using Minimap2. Viral species were considered positive only in the presence of ≥ 2 mapped reads or one mapped read longer than 400 bp. The haemagglutinin (HA) and neuraminidase (NA) subtype of IAV was determined based on the subtype of the reference sequence. Bacterial species accounting for > 1% of the total reads were also reported.

To recover as much consensus sequence as possible, another round of relaxed demultiplexing was performed, which required a barcode to be present at either end of each read and maximise the number of classified reads. We used relaxed demultiplexed data for the generation of consensus sequences only when the number of IAV reads per million reads, for samples within the same flow cell, differed by < 200 fold; otherwise, stringent demultiplexed data were used. This cutoff was selected as shown in Supplemental Figure S2. Nanopolish v0.9.2 [17] was used to detect single nucleotide (nt) variants and a consensus sequence was generated using the margin_cons.py script [9]. Finally, reads were mapped against the consensus sequence and only positions that were supported by ≥ 70% of reads were kept. Consensus sequences generated with Nanopolish were used for the following drug resistance and phylogenetic analyses. Sequence data have been uploaded to the European Nucleotide Archive under study accession number PRJEB45991.

### Drug resistance analysis

Resistance to antiviral agents (oseltamivir, zanamivir, and amantadine) was analysed using the consensus sequences of NA and Matrix protein 2 gene (M2). Drug resistance mutations are listed in Supplemental Table S2, based on a previously published list [18].

### Phylogenetic analyses

Phylogenetic trees were generated both for each gene segment of IAV and for the complete IAV genome. For each gene segment of IAV, an integrated dataset comprising our sequences (segment coverage > 50%) and a set of influenza reference sequences [19,20] was used. Segment-specific maximum-likelihood phylogenies were generated using RAxML v8.2.10 [21], in which a general time-reversible model of nt substitution and a gamma-distributed rate variation among sites was applied. Topological robustness of the tree was evaluated by 500 pseudo-replicates. Sequence alignments were performed using Multiple Sequence Alignment (MUSCLE) v3.8 [22]. For the complete IAV genome, we used our sequences (genome coverage > 70%) together with influenza A(H1N1)pdm09 (pH1N1) and seasonal H3N2 virus sequences (600 complete genomes each) circulating in Europe during the 2018/19 flu season, downloaded from Global initiative on sharing all influenza data (GISAID; Supplemental Table S3) [23]. Pairwise distance was calculated using only genome positions where both sequences possessed base-called nt. A minimum spanning tree for the complete genome was generated using igraph package in R [24].

### Ethical statement

The study of anonymised discarded clinical samples was approved by the London - Queen Square Research Ethics Committee (17/LO/1420).

## Results

### Nanopore sequencing of influenza directly from respiratory samples

Nanopore sequencing generated between 4.9 × 10^3^ and 4.1 × 10^6^ (mean: 4.3 × 10^5^) total reads per sample (Supplemental Table S1A). We retrieved Hazara virus reads (spiked as an internal control at 10^4^ genome copies/mL) from 147/180 (82%) samples. The 33 samples in which Hazara virus reads were not identified were all influenza negative and had comparatively low total cDNA concentrations following amplification. Therefore, we repeated sequencing of the 18/33 samples that had sufficient remaining material with the addition of linear polyacrylamide as a carrier to the extraction buffer, which produced Hazara virus reads in 16/18 samples. Taken together, we therefore retrieved Hazara internal control in 163/180 (91%) samples, ranging from one to 29,889 reads (Supplemental Table S1A).

### Identification, subtyping, and recovery of influenza A viral genomes

**Figure 2:**
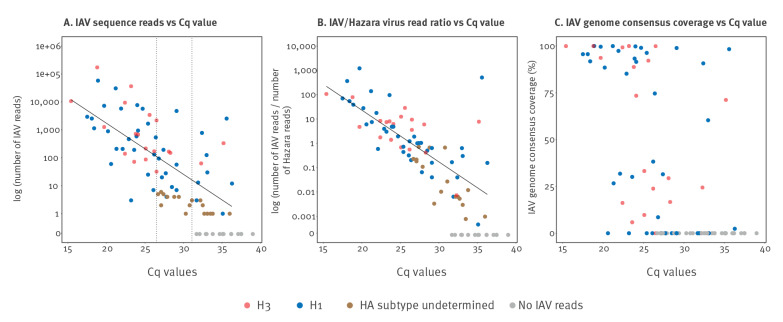
Identification, subtyping, and recovery of influenza A virus genomes by Nanopore metagenomic sequencing of respiratory samples that tested influenza-positive in a clinical diagnostic laboratory, United Kingdom, 2018/19 influenza season (n = 90 respiratory samples)

IAV: influenza A virus; Cq: quantification cycle; HA: haemagglutinin.

The Xpert Xpress Flu/RSV assay reported Cq values ranging from 15.4 to 39.0 (mean: 28.0) in the 90 influenza-positive samples, distributed across the influenza season (Figure 1A and 1B). Influenza subtyping results were not generated by routine clinical testing. We identified IAV reads in 75 of 90 influenza-positive samples (sensitivity 83%), ranging from one to 171,733 reads (Figure 2A). IAV reads were present in all 58 samples with Cq ≤ 31, and up to a maximum Cq value of 36.3 (sample 48, 12 IAV reads). There was a strong correlation between Cq value and both IAV read numbers (R^2^ = 0.43, p < 0.0001; Figure 2A) and the ratio of IAV:Hazara virus reads (R^2^ = 0.54, p < 0.0001; Figure 2B). The remaining 15 influenza-positive samples for which IAV reads were not generated by Nanopore sequencing had lower viral titres, reflected by higher Cq values (range: 31.7–39.0). IAV reads were not present in 84/90 influenza-negative samples (specificity 93%) (Supplemental Table S1A).

A. Number of IAV reads generated by Nanopore sequencing (Oxford Nanopore Technologies, Oxford, United Kingdom) against Cq value; R^2^ = 0.43, p < 0.0001. IAV reads were present in all samples with Cq ≤ 31 (right dashed line), and HA subtype was determined for all samples with Cq ≤ 27 (left dashed line).

B. Ratio of the number of IAV:Hazara virus reads against Cq value; R^2^ = 0.54, p < 0.0001. Hazara virus was spiked as an internal control at 10^4^ genome copies/mL.

C. Coverage of the IAV consensus sequence against Cq value.

Cq values were derived from the routine diagnostic test, which is the Xpert Xpress Flu/RSV assay (Cepheid, Sunnyvale, CA, United States).

We were able to determine the HA subtype of 59/90 (65%) samples; 40 were H1 and 19 were H3 (designated as blue vs red dots in Figure 2). We could determine HA subtype for all samples with Cq ≤ 27, and up to a maximum of Cq equal to 36.3 (sample 48) (Figure 2A). Subtyping results were not available for the remaining samples due to limited HA coverage. We retrieved 28/90 (31%) consensus sequences with genome coverage ≥ 70%, among which 18 were H1 and 10 were H3 subtype (Figure 2C). We retrieved 12/90 (13%) consensus sequences with genome coverage between 10% and 60%. The genome coverage for samples with Cq value between 20 and 25 showed substantial variation, which was not associated with any sample attributes that we were able to measure, including sample type, or percentage of human or bacterial reads (data not shown).

### Identification of drug-resistant mutations

From consensus sequences covering drug-resistant positions, we identified the S31N amino acid mutation in the M2 protein in 20/20 H1N1 and 11/11 H3N2 sequences, which is known to be widespread, conferring reduced inhibition by amantadine [25]. One of 13 H3N2 sequences (sample 5) carried the S331R amino acid mutation in the NA protein, which has been reported to confer reduced inhibition by oseltamivir [26]. Other drug resistance mutations, such as H275Y in the NA protein associated with oseltamivir resistance [27], were not present in our dataset.

### Identification of H3N2 reassortant influenza A virus

**Figure 3:**
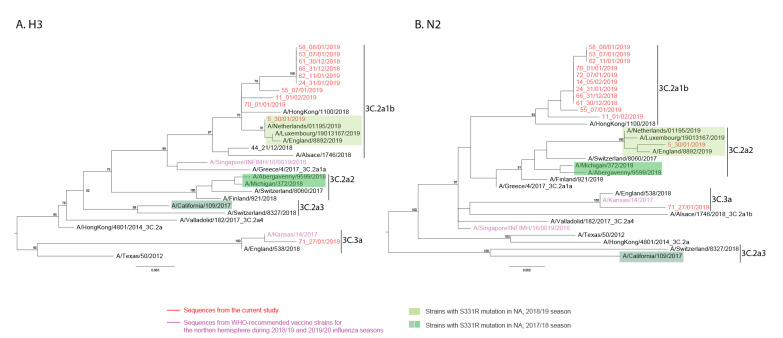
Maximum likelihood phylogenies of H3N2 influenza A virus sequences recovered from respiratory samples collected from a hospital cohort, United Kingdom, 2018/19 influenza season (n = 11 H3 sequences and 13 N2 sequences)

The majority of our H3 sequences were clustered within clade 3C.2a1b, with one sequence in clade 3C.3a (Figure 3A). Comparison of the H3 and N2 phylogenies showed that HA and NA segments of each individual sample were clustered within the same clade, except sample 5 had a distinct genotype with the H3 segment clustered within clade 3C.2a1b and the NA segment within clade 3C.2a2 (denoted subsequently as ‘R-genotype’), suggesting intra-subtype reassortment (Figure 3A and 3B).

WHO: World Health Organization.

A. H3 segment.

B. N2 segment.

Sequences recovered from this study are marked in red, vaccine seed strains recommended by WHO for the northern hemisphere 2018/19 and 2019/20 influenza season are in purple, and reference sequences representing major genetic clades of seasonal H3N2 are in black. Genetic clades are indicated on the right of the tree. Green boxes in different shades indicate sequences that carry the S331R mutation in the NA segment: the light green box represent sequences from the 2018/19 season, and two darker green boxes represent sequences from the 2017/18 season. Numbers at the nodes indicate bootstrap support values. The length of the branches in the tree are proportional to the number of nt substitutions per site as indicated by the scale.

Interestingly, the S331R mutation occurred in the same sample (sample 5), motivating us to further investigate the prevalence of this mutation in seasonal IAV using all global H3N2 sequences published in GISAID from the last two influenza seasons (2017/18 and 2018/19). In the 2017/18 dataset, 13/7,129 (0.2%) sequences carried the S331R mutation, with HA and NA segments from clade 3C.2a2 or 3C.2a3. In 2018/19, the proportion of sequences with the S331R mutation increased to 139/9,274 (1.5%), and all belonged to the R-genotype. These results suggest a potential association between the increase in prevalence of the S331R mutation and the emergence of this distinct R-genotype.

### Nosocomial transmission of H3N2 influenza A virus

**Figure 4:**
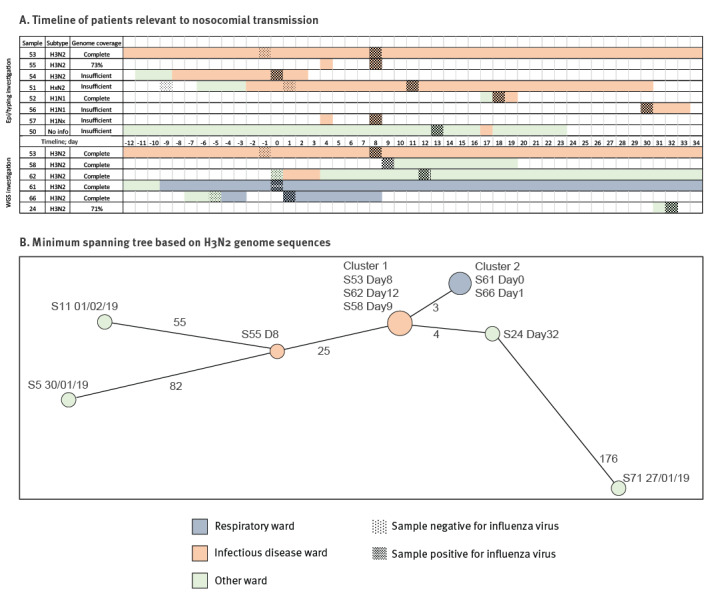
Investigation of nosocomial transmission of influenza A(H3N2) virus in a hospital, United Kingdom, 2018/19 influenza season (n = 14 patients)

We included a putative clinical cluster of eight influenza-positive samples (group 3) collected from patients on the infectious diseases ward over a 30 day period, aiming to investigate potential nosocomial transmission events (Figure 4A). We could determine the HA subtype of six samples, three being H3 and three H1. Among these, two H3N2 (samples 53 and 55) and one H1N1 consensus sequences had > 70% full genome coverage. A minimum spanning tree (MST) of our H3N2 sequences showed that samples 53 and 55 differed by 25 single nt polymorphisms (SNP)s (Figure 4B), despite being collected on the same day from patients on the ward. These results refuted the suspicion that these eight samples from the infectious diseases ward were all associated with a single nosocomial transmission cluster, and suggested that some, if not all, of the patients had acquired influenza infection independently. However, we were unable to define detailed relationships between these eight samples, due to insufficient retrieved genome sequences.

Epi: epidemiological; info: information; WGS: whole genome sequencing; SNP: single nucleotide polymorphism.

A. Timeline of patients relevant to nosocomial transmission. Each row represents one patient. Timeline indicated in days in the middle divides the plot into two parts: the top part represents eight patients from a putative transmission cluster on the infectious diseases ward, and the bottom part represents six patients with potential nosocomial transmission (differ by ≤ 4 SNPs) based on Nanopore sequencing (Oxford Nanopore Technologies, Oxford, United Kingdom) results.

B. Minimum spanning tree of H3N2 genomes (coverage > 70%). The tree was built on the basis of single nucleotide variant distances between consensus sequences. Distance between each pair of sequence is denoted by number adjacent to the branch.

The H3N2 MST (Figure 4B) also demonstrated that three sequences were identical (cluster 1), to that of one patient on the infectious diseases ward (sample 53), one who had been recently on the infectious diseases ward and then under the care of emergency assessment unit (EAU) (sample 62), and one who had been on the EAU for a couple of days and then in the complex medicine unit until discharged (sample 58). Furthermore, two identical sequences (cluster 2) differed from cluster 1 by 3 SNPs, and were from patients on the respiratory ward, taken 1 day apart. One sequence (sample 24) differed from cluster 1 by 4 SNPs, and was from an acutely admitted patient in the EAU 3 weeks later. The remaining four sequences, including sample 55 from the refuted cluster and three from patients elsewhere in the hospital, were separated from cluster 1, cluster 2, and each other by ≥ 25 SNPs.

These results suggested that cluster 1 patients connected to the infectious diseases ward and cluster 2 patients on the respiratory ward likely reflected nosocomial transmission. There was no clear epidemiological link between cluster 1 patients, cluster 2 patients, and the acutely admitted EAU patient (sample 24). One patient in cluster 1 (sample 58) and this EAU patient were positive for influenza within the 2 first days of their admission to the hospital, suggesting these samples may be associated with a predominant strain circulating in the community.

### Independent introductions of influenza A(H1N1)pdm09 viruses

Phylogenetic analysis of the H1 segment showed that our sequences clustered within clade 6B.1 (Figure S3A). At the full genome level, we found no evidence of phylogenetic clustering of pH1N1 IAVs recovered from our hospital, suggesting these represent independent introductions. Rather, our pH1N1 genomes were closely related to other genomes recovered during the UK 2018/19 season (Figure S3B). Twelve of our pH1N1 genomes had their most closely related sequence within < 15 SNPs, 11 of these most closely related sequences were from the UK.

### Pilot study of testing for five other respiratory viruses

Among the 90 influenza-negative respiratory samples we sequenced, 55 had tested positive for another virus in the clinical diagnostic laboratory. From this small dataset, our metagenomic sequencing dataset was > 80% sensitive for HMPV, RSV, and PIV, but only 30% sensitive for coronavirus and enterovirus; specificity was high at > 94% for all five viruses (Table).

**Table:** Summary of results for five respiratory viruses derived from Nanopore sequencing data of respiratory samples collected from a hospital cohort, United Kingdom, 2018/19 influenza season (n = 90 samples)

Virus	Number positive based on Biofire testing in clinical laboratory	True positive	False negative	Sensitivity %	True negative	False positive	Specificity %
Human metapneumovirus	5	4	1	80	83	2	98
Respiratory syncytial virus	11	9	2	82	75	4	95
Parainfluenza	11	9	2	82	77	2	97
Coronavirus	10	3	7	30	79	1	99
Enterovirus	20	6	14	30	68	2	97

Samples were tested in the clinical diagnostic laboratory using BioFire FilmArray Respiratory Panel 2 assay (BioFire Diagnostics, Salt Lake City, Utah, United States) for a panel of respiratory pathogens. True and false positive and negative results pertain to results of Nanopore sequencing (Oxford Nanopore Technologies, Oxford, United Kingdom).

### Identification of reads from organisms not tested for in the clinical laboratory

In five influenza-positive samples for which IAV reads were generated by sequencing, we also retrieved reads for other viruses, including human coronavirus HKU1 (sample 17, 996 reads covering the complete genome; sample 14, 76 reads), human PIV3 (sample 40, three reads), rhinovirus A (sample 10, one read), and classical human astrovirus (sample 19, one read, 462 bp) (Supplemental Table S1B). For the 90 influenza-negative samples, sequencing data did not show reads likely to represent viral pathogens other than those already identified by BioFire RP2.

From our complete collection of 180 samples, we identified reads from five bacterial species, *Streptococcus pneumoniae* (n = 37 samples), *Pseudomonas aeruginosa* (n = 5), *Moraxella catarrhalis* (n = 3), *Staphylococcus aureus* (n = 1), and *Haemophilus influenzae* (n = 1). While these organisms may represent agents of respiratory infection, they can also be commensal or colonising flora. In the absence of detailed clinical metadata, we were unable to explore their likely contribution to pathology.

## Discussion

### Turnaround time for metagenomic sequencing

In this study, we conducted Nanopore metagenomic sequencing of IAV directly from clinical respiratory samples at a UK hospital during the 2018/19 influenza season, reporting a comparison with routine clinical diagnostic tests. The total turnaround time for metagenomic sequencing of each sample was < 72 h. While the turnaround time is still slower than other laboratory diagnostic tests, and the approach requires an investment in labour and sequencing/analysis time, there is potential to reduce this further, through simplification of the wet laboratory and bioinformatic protocols. The use of automated extraction systems with column-based genomic DNA removal, development of a cartridge-based SISPA system and use of the rapid Nanopore sequencing kit, along with implementation of live base-calling, shorter run times and real-time bioinformatic analysis of reads, would greatly improve turnaround and handling times for routine use in a clinical laboratory setting. However, run times would still need to be long enough to generate a sufficient number of reads to detect low titre samples and produce a consensus sequence.

Timeliness is crucial for the deployment of international vaccine strategies. Each February, the World Health Organization (WHO) determines influenza vaccines for use in the following northern hemisphere influenza season. However, in 2019, WHO postponed the vaccine update until late March to include a clade 3C.3a H3N2 strain (Supplemental Figure S1), due to the substantial increase of 3C.3a viruses in several WHO regions since November 2018 associated with low vaccine effectiveness (5%) [28]. This one-month delay raised concerns about the timeliness of vaccine manufacturing and distribution for the upcoming influenza season. Within our cohort, a clade 3C.3a H3N2 sample was collected on 27 January 2019, and if we had conducted rapid-turn-around sequencing as a routine assay then the complete genome sequence could be available in < 72 h. This timeline illustrates how routine laboratory sequencing would allow timely genetic characterisation, providing translational advantages in influenza surveillance, monitoring change in the proportion of genetically diverse strains, and contributing to timely insights into seasonal epidemiology vaccine design.

### Sensitivity of Nanopore metagenomic sequencing

Our sequencing data showed 83% sensitivity for IAV compared with existing laboratory diagnostic tests, consistent with our previous study [13]. Further optimisation is needed to improve the sensitivity of our protocol for samples with lower viral titres (Cq values > 30). Potential methods include depletion of host and bacterial RNA to reduce the amount of non-target nucleic acid present. However, such methods can be expensive and would increase turnaround time. Amplicon-based sequencing is an alternative approach to generate IAV sequences, which would improve the sensitivity of the assay. However, such an approach detects only the target pathogen, without the benefit of identifying a broad range of respiratory pathogens provided by metagenomic approaches.

Samples in which Hazara virus reads were not identified were all IAV negative and had comparatively low total cDNA concentrations following amplification, suggesting low viral titres in these samples. However, some of these samples were positive for other respiratory viruses. Our data show that addition of a carrier can improve the detection of internal spiked control in samples with low total cDNA, which is likely due to the improved purification and reduced degradation of lower concentration RNA, thus we intend to incorporate this approach as a routine part of the protocol in future.

Our data showed a large range in the number of total reads generated per sample (from 4.9 × 10^3^ to 4.1 × 10^6^). All flow cells were loaded with the recommended amount of sequencing library. The observed number of total sequencing reads did not show a correlation with Cq value of the sample. These data suggest that further improvements are needed for Nanopore sequencing technology to be reliably used as a quantitative assay.

Low numbers of IAV reads were identified in influenza-positive samples with high Cq values, as well as six influenza-negative samples. Based on the current performance of Nanopore sequencing and barcode demultiplexing, it can be difficult to distinguish between samples with low viral titre and possible contamination.

In order to mitigate problems of cross barcode contamination, IAV positive samples were batched by Cq value and all samples were initially demultiplexed using stringent criteria. However, the application of stringent barcode demultiplexing leads to a considerable reduction in available data for each sample [29]. When multiplexing clinical samples, read numbers need to be corrected for background or be sufficiently distinct from other samples processed to rule out contamination. In a clinical setting, it would therefore, be preferable to sequence samples individually on a low input flow cell, e.g. ONT Flongle, to avoid these problems, give higher confidence in samples with low IAV read numbers and potentially improve the sensitivity for clinical samples with a lower viral titre (Cq ≥ 30). Further work on the production of a metabiome dataset from healthy controls would be of great value and give an insight to the clinical significance of viral reads.

There is a correlation between the number of IAV reads and genome coverage from our data. However, the genome coverage for samples with Cq value between 20 and 25 showed substantial variation. The lower than expected number of IAV reads for some samples is not accounted for by sample type or the level of background human or bacterial reads. Samples were stored at − 80 °C after receipt in the laboratory, however, delays in sample processing before receipt could potentially have an impact on sample integrity.

### Drug resistance

The S331R NA mutation in H3N2 IAV has been associated with reduced susceptibility to oseltamivir [26,30,31]. Among 1,039 H3N2 IAVs tested globally during the 2018/19 season, one strain from South Korea showed reduced susceptibility to oseltamivir because of this mutation [32]. Our analysis demonstrates that IAVs carrying this mutation can belong to a distinct genotype generated through intra-subtype reassortment between clades 3C.2a1b and 3C.2a2. A previous study reported a similar observation that the emergence and rapid global spread of adamantane resistant H3N2 IAVs (conferred by a S31N mutation in the Matrix protein 2) was associated with a single genotype generated through intra-subtype reassortment [33,34]. S31N now occurs in almost all circulating IAV globally, causing the cessation of use of adamantane to treat influenza [25]. The genesis, prevalence, distribution and clinical impact of the S331R mutation merits additional study to evaluate potential implications for the clinical usefulness of oseltamivir, which is widely used as a first-line agent when treatment is indicated [30].

### Mapping outbreaks and transmission

Whole genome sequencing can provide high resolution characterisation of the spatiotemporal spread of viral outbreaks [7,8]. Previous studies have used targeted enrichment combined with next generation sequencing to investigate nosocomial transmission of influenza [35,36], and our study demonstrates the application of Nanopore metagenomic sequencing for this purpose. Our sequencing data allow us to refute the suspicion of a single transmission cluster on the infectious diseases ward, although the small number of whole genomes generated limits the extent to which we could draw conclusions about transmission among this specific patient group. Furthermore, our dataset reveals two clinical clusters that likely represent nosocomial transmission, showing proof of concept that Nanopore metagenomic sequencing can identify transmission, and inform infection prevention and control practices.

### Detection of organisms other than influenza A virus

Based on a very small exploratory dataset, our protocol shows > 80% sensitivity for the detection of HMPV, PIV, and RSV compared with routine clinical diagnostic testing. The lower sensitivity for enterovirus and coronavirus could be due to low viral titres in these samples, although we are not able to confirm this as the BioFire RP2 assay is a non-quantitative test. Another possibility is that the SISPA method is less sensitive for certain viruses [37]. Moreover, no influenza B virus reads were present in our 90 influenza-positive samples, congruent with the global low level of influenza B virus during the 2018/19 season. Further work is needed to determine the limits of detection and optimise the laboratory and bioinformatic protocol to improve the sensitivity.

### Comparison with Illumina sequencing

The Illumina sequencing platform is widely regarded as the gold-standard of sequence quality. Data from our recent work have shown that the proportion of influenza reads generated from Nanopore and Illumina metagenomic sequencing is very similar [13], therefore, the limit of detection is likely to be very close for these two platforms.

Nanopore sequencing data are characterised by high error rates on the sequencing read level (ca 10% with R9.4 chemistry and current base-calling algorithms), particularly in homo-polymeric regions. However, the largely random nature of the error, in combination with adequate sequencing depth would allow Nanopore sequencing to generate consensus sequences of similar accuracy to Illumina. We have previously reported a comparison between consensus sequences derived by Nanopore and Illumina for 15 IAV and 16 HMPV positive clinical respiratory samples, and found that, at a sequencing depth cutoff of 10×, Nanopore consensus sequences were 99.95–100% identical to Illumina sequences [12,13]. Additionally, the high error rate of Nanopore reads is compensated by the increased read length, which could provide an advantage in individual read taxonomic assignment.

### Implications for public health

Nanopore metagenomic sequencing of full-length viral genome sequences directly from clinical samples has many potential applications in public health settings, including routine surveillance or during emerging outbreaks. On-site and real-time sequencing with ONT devices enables timely identification of an outbreak. Nanopore metagenomic sequencing of the severe acute respiratory syndrome coronavirus 2 (SARS-CoV-2) has provided critical and timely evidence for human-to-human transmission of this virus [38].

At present, most diagnostic laboratories do not generate any information about drug resistance. Specimens must be submitted to regional or diagnostic reference laboratories for this investigation. Thus, in-house methods that combine the potential for diagnosis together with drug resistance offer a significant advance over existing standards of care, albeit requiring further improvements in turnaround time, genome coverage and read depth.

### Caveats and limitations

This study included a limited cohort, with samples stratified by clinical diagnostic results, collection time, and the observation of a putative clinical cluster. We were not able to systematically sequence all influenza-positive samples from the clinical diagnostic laboratory due to limited staff and laboratory resources. Generalisability is limited by this sampling approach, as well as by other confounding influences which we were unable to control (e.g. diverse sample types, sample exposure to freeze/thawing, underlying immunocompromise, symptom duration before sample collection).

While metagenomic data hold the promise for simultaneous detection of all pathogens from an individual clinical sample, they pose general challenges to interpretation of the results. Running parallel negative controls and samples from healthy controls would provide insights into identification of potential contaminants and help move towards drawing distinctions between pathogens and commensal flora.

There are not yet enough data to know what thresholds should be set for calling a clinical sample ‘positive’ or ‘negative’ based on Nanopore data alone. More data are needed on clinical phenotypes of disease, clinical laboratory diagnostic results, and attributes of metagenomic data (read numbers, sequencing depth, read lengths, and genome coverage) to determine the right thresholds for diagnostic calling. These thresholds may vary between sample types, pathogens, and disease phenotypes. As well as positive/negative diagnosis, there could be equivocal categories in which relevant read information has been generated, but do not meet the thresholds determined for diagnosis.

## Conclusions

Nanopore sequencing can be applied in clinical settings to simultaneously detect influenza and other respiratory viruses, identify drug resistance mutations, characterise genetic diversity, and investigate potential nosocomial transmission events. While work is still needed to refine and streamline the sequencing protocol and bioinformatic analysis, Nanopore metagenomic sequencing has the potential to become an applicable point-of-care test for infectious diseases in clinical settings.

## Data availability

Following removal of human reads, our sequencing data have been uploaded to the European Bioinformatics Institute https://www.ebi.ac.uk/, project reference PRJEB.

## Supplementary Data

254000 Supplementary Methods and Figures

## Supplementary Data

41000 Supplementary Tables S1 and S2

## Supplementary Data

307000 Supplementary Table S3
