# Supplementary material for: Nanopore metagenomic sequencing of influenza virus directly from respiratory samples: diagnosis, drug resistance and nosocomial transmission, United Kingdom, 2018/19 influenza season
Source: Euro Surveill. 2021 Jul 8;26(27):2000004. doi: 10.2807/1560-7917.ES.2021.26.27.2000004 (PMC8268652; doi:10.2807/1560-7917.ES.2021.26.27.2000004)
Supplement: Supplementary Methods and Figures [file FINAL_2000004_XU_Suppl_Methods_Figs.pdf]

This supplementary material is hosted by Eurosurveillance as supporting information alongside the article [Nanopore metagenomic sequencing of influenza virus directly from respiratory samples: diagnosis, drug resistance and nosocomial transmission, United Kingdom, 2018/19 influenza season], on behalf of the authors, who remain responsible for the accuracy and appropriateness of the content. The same standards for ethics, copyright, attributions and permissions as for the article apply. Supplements are not edited by Eurosurveillance and the journal is not responsible for the maintenance of any links or email addresses provided therein.

SUPPLEMENTARY METHODS

Sample processing for sequencing

180 samples were processed as described in detail previously [1]. Briefly, viral transport media from samples, spiked with 10^4 genome copies per ml Hazara virus as a positive internal control, was centrifuged to remove bacteria and cellular debris. Total nucleic acid was extracted, DNA was removed enzymatically and the remaining RNA was randomly reverse transcribed and amplified using sequence independent single primer amplification (SISPA). The resulting cDNA was prepared for Nanopore sequencing.

Nanopore library preparation and sequencing

Multiplex sequencing libraries were prepared using 200fmol of cDNA from six samples as input to the SQK-LSK109 Kit, barcoded individually using the EXP-NBD104 Native barcodes (Oxford Nanopore Technologies, ONT). Libraries were sequenced on FLO-MIN106 flow cells on a GridION device (ONT), with sequencing proceeding for 48h. Samples for which sequencing did not produce reads mapping to the internal control Hazara virus genome were associated with low quantities of total cDNA and so were

repeated with the addition of 5 µg Linear polyacrylamide carrier (Thermo Fisher) to the AVL lysis buffer (Qiagen).

In order to evaluate how feasible it would be to run this methodology in ‘real-time’, we aimed to sequence as many of the 180 samples as possible in the first week after starting laboratory work. A workflow was created with different team members performing each stage of the sample processing, Nanopore library preparation and sequencing.

Genomic analysis

Nanopore reads were basecalled using Guppy v3 (ONT). Human reads were removed using CRuMPIT workflow [2]. In order to minimize the number of misclassified reads and to allow accurate identification of viral species, stringent barcode demultiplexing was performed, which required the same barcode to be present at both ends of each read, using Porechop (v0.2.2, https://github.com/rrwick/Porechop). Reads were taxonomically classified against the RefSeq database using Centrifuge v1.0.3 [3]. Reads were then mapped using Minimap2 [4] to a reference genome for each viral species identified by Centrifuge; a draft consensus sequence for each species was generated by a simple majority voting method. This resulting draft consensus sequence was BLASTed against a custom database containing genomes of influenza, coronavirus, HMPV, RSV, parainfluenzae, and enterovirus, to identify the closest reference genomes. Reads were then mapped against the identified reference genome using Minimap2. Viral species were considered positive only in the presence of ≥2 mapped reads or one mapped read longer than 400bp. The HA and NA subtype of IAV was determined on the basis of the subtype of the reference sequence. Bacterial species accounting for >1% of the total reads were also reported.

To recover as much consensus sequence as possible, another round of relaxed barcode demultiplexing was performed, which required a barcode to be present at either end of each read and maximize the number of classified reads. Nanopolish v0.9.2 [5] was used to detect single nucleotide variants and a consensus sequence was generated using the margin_cons.py script [6]. Finally, reads were mapped against the consensus sequence and only positions that were supported by ≥70% of reads were kept.

Drug resistance analysis

Resistance to antiviral agents (oseltamivir, zanamivir, and amantadine) was analyzed using the consensus sequences of Neuraminidase and Matrix 2 gene. Drug resistance mutations from [7] were listed in Table S2.

Phylogenetic analyses

Phylogenetic analyses were conducted as follows:

(i) for each gene segment of IAV using an integrated dataset comprised of our sequences (segment coverage >50%) and a set of influenza reference sequences [8,9].

(ii) for the complete IAV genome using our sequences (genome coverage >70%) together with pH1N1 and seasonal H3N2 viruses (600 complete genomes each) circulating in Europe during the 2018/1[9]9 flu season, downloaded from Global initiative on sharing all influenza data (https://www.gisaid.org) [10].

Maximum-likelihood phylogenies were generated using RAxML v8.2.10 [11], in which a general time-reversible model of nucleotide substitution and a gamma-distributed rate variation among sites was applied. Sequence alignments were performed using MUSCLE v3.8 [12]. A minimum spanning tree was generated using igraph package in R.

Figure S1. Subtype of influenza A virus determined by Nanopore metagenomic sequencing for 90 respiratory samples from a UK tertiary referral hospital during the 2018/19 influenza season.

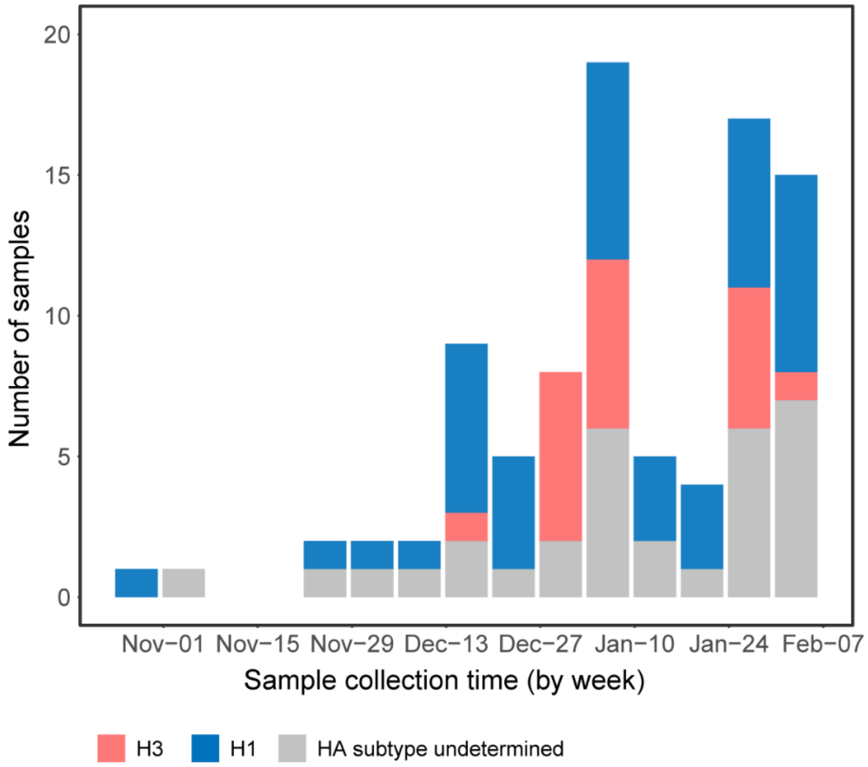

Figure S2. Maximum likelihood phylogenies of H1N1 sequences recovered from respiratory samples collected from patients in a UK tertiary referral hospital during the 2018/19 influenza season. A) H1 gene segment sequences recovered from this study are marked in blue, vaccine seed strains recommended by WHO for the northern hemisphere 2018/19 and 2019/20 influenza season are in yellow, and reference sequences represent major genetic clades of H1N1 IAV are in black. The clade 6B.1 are indicated on the right of the tree. B) H1N1 complete genome, the most closely related strain for each of our sequence (in black) were selected from 600 pH1N1 IAVs circulated in European countries during 2018/19 influenza season.

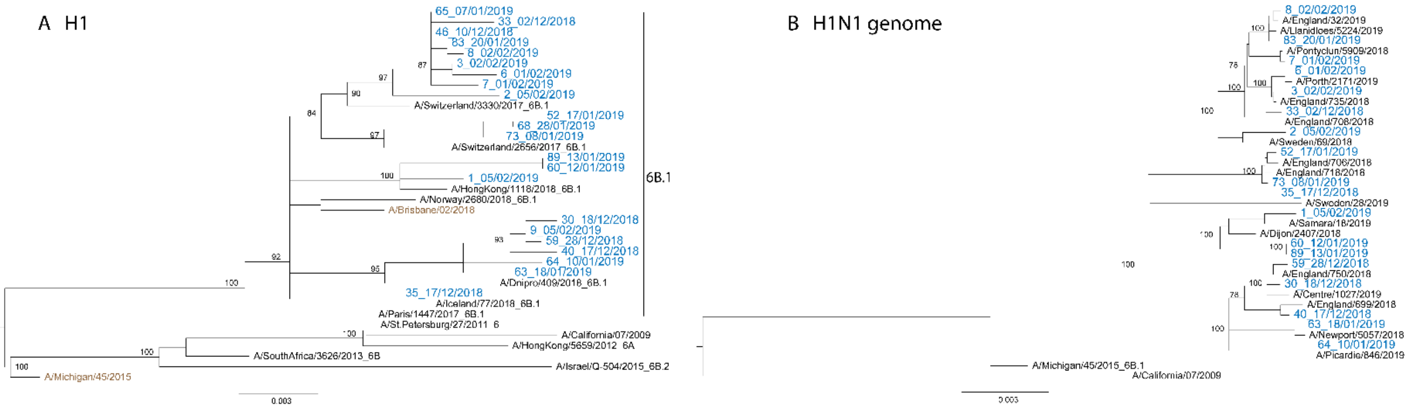

Table S1 Summary of Nanopore sequencing data derived from 180 respiratory samples collected from a UK hospital cohort during 2018/19 influenza season.

Table S2 List of mutations that confer reduced inhibition by oseltamivir, zanamivir, and amantadine.

REFERENCES

1. Lewandowski K, Xu Y, Pullan ST, Lumley SF, Foster D, Sanderson N, et al. Metagenomic Nanopore sequencing of influenza virus direct from clinical respiratory samples. doi:10.1101/676155

2. Sanderson ND, Street TL, Foster D, Swann J, Atkins BL, Brent AJ, et al. Real-time analysis of nanopore-based metagenomic sequencing from infected orthopaedic devices. BMC Genomics. 2018;19: 714.

3. Kim D, Song L, Breitwieser FP, Salzberg SL. Centrifuge: rapid and sensitive classification of metagenomic sequences. Genome Res. 2016;26: 1721–1729.

4. Li H. Minimap2: pairwise alignment for nucleotide sequences. Bioinformatics. 2018;34: 3094–3100.

5. Loman NJ, Quick J, Simpson JT. A complete bacterial genome assembled de novo using only nanopore sequencing data. Nat Methods. 2015;12: 733–735.

6. Quick J, Grubaugh ND, Pullan ST, Claro IM, Smith AD, Gangavarapu K, et al. Multiplex PCR method for MinION and Illumina sequencing of Zika and other virus genomes directly from clinical samples. Nat Protoc. 2017;12: 1261–1276.

7. Van Poelvoorde LAE, Saelens X, Thomas I, Roosens NH. Next-Generation Sequencing: An Eye-Opener for the Surveillance of Antiviral Resistance in Influenza. Trends Biotechnol. 2019. doi:10.1016/j.tibtech.2019.09.009

8. Meijer A, Swaan CM, Voerknecht M, Jusic E, van den Brink S, Wijsman LA, et al. Case of seasonal reassortant A(H1N2) influenza virus infection, the Netherlands, March 2018. Euro Surveill. 2018;23. doi:10.2807/1560-7917.ES.2018.23.15.18-00160

9. Wiman Å, Enkirch T, Carnahan A, Böttiger B, Hagey TS, Hagstam P, et al. Novel influenza A(H1N2) seasonal reassortant identified in a patient sample, Sweden, January 2019. Eurosurveillance. 2019. doi:10.2807/1560-7917.es.2019.24.9.1900124

10. GISAID - Global Initiative on Sharing All Influenza Data. [cited 3 Nov 2019]. Available: https://www.gisaid.org

11. Stamatakis A. RAxML version 8: a tool for phylogenetic analysis and post-analysis of large phylogenies. Bioinformatics. 2014;30: 1312–1313.

12. Edgar RC. MUSCLE: multiple sequence alignment with high accuracy and high throughput. Nucleic Acids Research. 2004. pp. 1792–1797. doi:10.1093/nar/gkh340
